# Half-Metallic Properties of Single-Walled Polymeric Manganese Phthalocyanine Nanotubes

**DOI:** 10.3390/s120708438

**Published:** 2012-06-25

**Authors:** Hongbin Jiang, Meilin Bai, Peng Wei, Lili Sun, Ziyong Shen, Shimin Hou

**Affiliations:** 1 School of Physics & Electronic Engineering, Sichuang Mianyang Normal University, Mianyang 621000, China; E-Mail: 271479861@qq.com; 2 Key Laboratory for the Physics and Chemistry of Nanodevices, Department of Electronics, Peking University, Beijing 100871, China; E-Mails: meilin.bai@gmail.com (M.B.); weipeng.cumt@163.com (P.W.); sunlili.cb@gmail.com (L.S.); zyshen@pku.edu.cn (Z.S.)

**Keywords:** MnPc nanotubes, half-metal, sensor, molecular spintronics, density functional theory

## Abstract

We present a theoretical study of the electronic and magnetic properties of single-walled manganese phthalocyanine (MnPc) nanotubes which can be thought of as rolled-up ribbons of the two-dimensional (2D) polymeric MnPc sheet. Our density functional theory calculations show that all of the MnPc nanotubes investigated here are half-metals with 100% spin polarization around the Fermi level. Following the increase of the tube diameter, the number of spin-down energy bands of MnPc nanotubes is always increased while the spin-up band gap of MnPc nanotubes approaches that of the 2D MnPc sheet in an oscillatory manner. Because the half-metallic character of MnPc nanotubes is deeply rooted in the distribution of electrons in the energy bands dominated by the Mn 3d atomic orbitals, adsorption of CO molecules on the Mn ions leads to a redistribution of electrons in the Mn 3d orbitals and thus can tune precisely the spin state and electronic transport properties of MnPc nanotubes, demonstrating promising applications of MnPc nanotubes in future molecular spintronics and single-molecule sensors.

## 1. Introduction

Since their discovery in 1993 [1,2], single-walled carbon nanotubes (SWCNTs) have received considerable attention due to their promising applications in nanoelectronic devices [3,4]. Depending on their chirality and diameter, metallic and semiconducting SWCNTs can be used as interconnects and conducting channels, respectively. More recently, the applications of SWCNTs have been extended to molecular spintronics [5,6]. Since infinite, pristine SWCNTs are spin degenerate, spin polarization in SWCNT-based molecular spintronic devices is always generated either by magnetic dopants decorating the tube wall [7–9] or by magnetic molecules drafted on the tube sidewall or connected to the tube end [10–14]. However, it is rather difficult to control precisely the dopant positions in the tube sidewall and the nanotube-molecule coupling. Thus, it is highly desirable to find other tubular nanostructures with intrinsic spin polarization functionalities.

Because SWCNTs can be considered as formed by rolling up the graphene sheet into a cylinder, it is a good start for the search for new tubular nanostructures from some known two-dimensional (2D) atomic sheets with novel magnetic properties. One promising candidate is the 2D polymeric metallophthalocyanine (MPc) sheets wherein the incorporated transition metal atoms can feature multiple functional properties, depending on their specific type and coordination environments [15,16]. For example, the 2D FePc polymer, which has been synthesized on both metal (Au and Ag) surfaces and a thin insulting film [15], is predicted to be an antiferromagnetic semiconductor [16]; in contrast, the ground state of the 2D polymeric MnPc sheet is determined to be half-metallic with 100% spin polarization around the Fermi level [16]. To be specific, a metallic feature appears in the spin-down channel of the 2D polymeric MnPc sheet while the spin-up channel remains semiconducting. Therefore, in this paper we investigate the electronic and magnetic properties of single-walled MnPc nanotubes which are constructed from the MnPc sheet by cutting along the (100) direction. The calculation employs spin density functional theory (DFT) within the generalized gradient approximation (GGA). Our calculations show that, different from other nanotubes such as Si, SiC, BN, AlN, GaN and InN which are spin degenerate [17–22], the single-walled MnPc nanotubes investigated here are all half-metals. Following the increase of the tube diameter, the number of spin-down energy bands across the Fermi level is always increased. In contrast, the size dependence of the spin-up band gap is a little complex. Although the spin-up band gap of MnPc nanotubes approaches that of the 2D MnPc sheet as the tube diameter is increased, the gap values of MnPc nanotubes with an odd number of MnPc monomers per unit cell is larger than those with an even number of MnPc monomers. Fine tuning of the electronic and magnetic properties of MnPc nanotubes are illustrated by adsorption of CO molecules on the Mn ions, which demonstrates that MnPc nanotubes have promising applications in molecular spintronic devices and single-molecule sensors.

## 2. Calculation Method

In this work we use the SIESTA code to study the atomic and electronic structures of single-walled MnPc nanotubes [23]. SIESTA is an efficient numerical implementation of DFT, in which improved Troullier-Martins pseudopotentials are used to describe the atomic cores and the GGA functional in the form proposed by Perdew, Burke and Ernzerhof (PBE) is used to account for the electron-electron interactions [24,25]. The pseudopotentials are generated using the following electronic configurations: H, 1s^1^; C, 2s^2^2p^2^; N, 2s^2^2p^3^; O, 2s^2^2p^4^; Mn, 4s^1^3d^6^. The wave functions for the valence electrons are expanded over a finite range numerical basis set and a user-defined double-zeta plus polarization (DZP) basis set is constructed for all elements including hydrogen, carbon, nitrogen, oxygen and manganese [13,14]. Geometry optimization is performed by conjugate gradient until the forces are smaller than 0.03 eV·Å^−1^. The 2D MnPc sheet is placed in the xOy plane while the MnPc nanotubes are placed along the z axis. In order to model isolated MnPc sheets and nanoutbes, the distance between neighboring MnPc sheets (nanotubes) are set to be larger than 15 Å.

## 3. Results and Discussion

**Figure 1.**
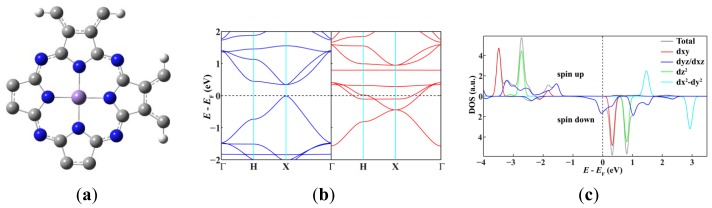
The optimized geometry structure of one unit cell (**a**) and the spin-resolved band structure (**b**) of the 2D MnPc sheet; (**c**) DOS projected onto the Mn 3d atomic orbitals.

Since single-walled MnPc nanotubes can be thought of as rolled-up ribbons of 2D MnPc sheets, we start from the atomic and band structures of the 2D polymeric MnPc sheet to understand the electronic and magnetic properties of MnPc nanotubes. As shown in Figure 1(a), the 2D MnPc polymer is a planar sheet with a square lattice, the lattice constant is calculated to be 10.71 Å; the Mn-N bond length is optimized to be 1.954 Å, very close to that of the gas-phase MnPc molecule [13]. In Figure 1(b,c) we present the spin-resolved band structure of the 2D polymeric MnPc sheet and the density of states (DOS) projected onto the Mn 3d atomic orbitals. As we can see, the 2D MnPc sheet has the electronic structure of a half-metal. For the spin-up electrons, the energy bands contributed by the π and π* orbitals of the Pc ring form respectively the valence band and the conduction band around the Fermi level, giving a direct gap (at the X point) of 0.36 eV, while the energy bands dominated by the Mn 3d orbitals are all far from the Fermi level. In contrast, for the spin-down electrons, two energy bands dominated by the Mn 3*d_xz_* and 3*d_yz_* orbitals cut across the Fermi level, resulting in a metallic character. The half-metallicity and ferromagnetism of the 2D MnPc sheet can be explained by a charge-transfer mechanism. In brief, although an isolated Mn atom has an [Ar]3d^5^4s^2^ electronic configuration, in the 2D MnPc sheet the Mn 4s levels are shifted above the Fermi level, and the strong crystalline field of D_4h_ symmetry splits the 3d shell into a doublet of e_g_ (*d_xz_*, *d_yz_*) symmetry and three singlets of a_1g_ (*d*_z^2^_), b_2g_ (*d_xy_*) and b_1g_ (*d*_x^2^−y^2^_) symmetry. Two of the seven valence electrons are transferred from the Mn atom to the Pc ring, and thus the central Mn ion has a 2+ formal charge. Four spin-up electrons completely fill the energy bands dominated by the Mn 3*d*_xy_, 3*d*_z^2^_, 3*d_xz_* and 3*d_yz_* orbitals, which hence are placed below the Fermi level. The remaining one valence electron only partially occupies the spin-down energy bands dominated by the Mn 3*d_xz_* and 3*d_yz_* orbitals. Consequently, the spin-down energy bands cross the Fermi level and the 2D MnPc sheet acquires a magnetic moment of 3.0 μ_B_ per unit cell, which is the same as that of an isolated MnPc molecule [13,14]. That is, polymerization does not change the spin state of the central Mn ion. These results obtained by using localized atomic orbitals are in good agreement with previous plane-wave DFT calculations [16]. It should be noted that the GGA + U method does not change the half-metallic characteristics of the 2D MnPc sheet though a very small in-plane shift occurs for the Mn ion [16].

**Figure 2.**
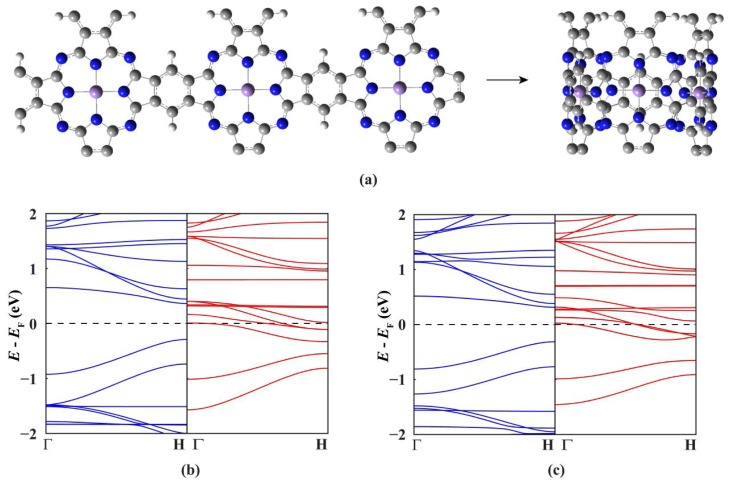
The optimized geometry structure of one unit cell (**a**) and the spin-resolved band structure (**c**) of the MnPc-3 nanotube, the spin-resolved band structure (**b**) of the MnPc nanoribbon with the same number of MnPc monomers along its width are given for comparison.

Now we move to investigate the electronic and magnetic properties of single-walled MnPc nanotubes. For the sake of brevity, these nanotubes are denoted by MnPc-n where n is the number of MnPc monomers in one unit cell. Figure 2(a) shows the optimized geometric structure of the thinnest MnPc-3 nanotube. Because the radius of the MnPc-3 nanotube is only 5.05 Å, the Mn-Mn distance perpendicular to the tube axis is calculated to be 8.642 Å which is about 2.07 Å shorter than that along the tube axis, demonstrating the large curvature of the tube wall. In order to know the effects of curvature of the tube wall, we compare the spin-resolved band structure (Figure 2(c)) of the MnPc-3 nanotube with that of the MnPc nanoribbon with the same number of MnPc monomers along its width (Figure 2(b)).

As we can see, the differences in the overall shape of these two band structures are very small, illustrating that the large curvature of the MnPc-3 tube wall does not affect the spin state of the Mn ions significantly. This is also corroborated by the magnetic moment per unit cell of the MnPc-3 nanotube (9.0 μ_B_), which is just three times of that of the 2D MnPc sheet. The most striking feature is that the MnPc-3 nanotube not only preserves the half-metallic characteristics but also has a larger spin-up band gap (0.63 eV) than the 2D MnPc sheet. For the spin-down electrons, among the six energy bands dominated by the Mn 3*d_xz_* and 3*d_yz_* orbitals five bands go across the Fermi level while another one approaches the Fermi level from higher energies at the H point. Thus, the ballistic transport for the infinite MnPc-3 nanotubes would be characterized by a conductance of 5*e*^2^/*h* for one of the spin channels and zero transmission for the other.

Next we investigate the size effects in MnPc nanotubes. Some important parameters characterizing the atomic and electronic structures of MnPc nanotubes are listed in Table 1, where the number of MnPc monomers per unit cell ranges from 3 to 6. As we can see, all four of these MnPc nanotubes are half-metals. Unlike armchair SWCNTs which all possess two energy bands going across the Fermi level [26], the number of the spin-down energy bands of MnPc nanotubes cutting the Fermi level always increases as the tube diameter is increased. In contrast, following the increase of the tube diameter the spin-up band gap of the MnPc nanotubes does not monotonically approach that of the 2D MnPc sheet, because the spin-up band gaps of MnPc nanotubes with an odd number of MnPc monomers in one unit cell are larger than those with an even number of MnPc monomers. The stability and the synthetic possibility of the MnPc nanotubes can be evaluated by the strain energy which is determined by subtracting the total energy per atom of the 2D MnPc sheet from the corresponding values of MnPc nanotubes. As listed in Table 1, the thermodynamic stability of MnPc nanotubes increases with the increase of the tube diameter, and MnPc nanotubes with diameters larger than the MnPc-6 nanotube will have much lower strain energies (<8 meV). That is, these MnPc nanotubes should be stable and can be rolled up easily from the 2D MnPc sheet. Although MnPc nanotubes have not been observed experimentally, our calculations indicate it is highly possible to prepare these MnPc nanotubes under appropriate experimental conditions considering that the 2D FePc sheet with similar structures has already been synthesized successfully [15].

**Figure 3.**
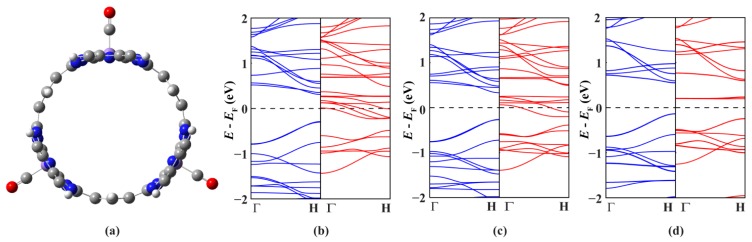
(**a**) the optimized geometry structure of one unit cell of the MnPc-3 nanotube adsorbed with three CO molecules, and the spin-resolved band structures of the MnPc-3 nanotube adsorbed with one (**b**); two (**c**) and three (**d**) CO molecules.

Finally we investigate how to control and tune the electronic and magnetic properties of MnPc nanotubes. It is well known that the spin state of metal phthalocyanine molecules can be tuned by ligand adsorption. For example, the spin on the FePc molecule can be quenched completely or partially, depending on whether the ligand bonded to the iron ion is CO or NO [27,28]. Therefore, we choose the MnPc-3 nanotube as a representative example and study the response of MnPc nanotubes to the adsorption of CO on the Mn ions (Figure 3). The adsorption of one or two CO molecules does not destroy the half-metallic character of the MnPc-3 nanotube, but does decrease the number of spin-down energy bands across the Fermi level. In details, only four spin-down energy bands can go across the Fermi level when one of the three Mn ions per unit cell of the MnPc-3 nanotube is bonded to a CO molecule, and the adsorption of one more CO molecule on another Mn ion further decreases the number of spin-down energy bands across the Fermi level to two. Correspondingly, the magnetic moment per unit cell is also decreased from 9.0 μ_B_ (pristine) to 7.0 μ_B_ (one CO molecule) and 5.0 μ_B_ (two CO molecules), indicating a change of the spin state of the Mn ions. However, when the three Mn ions in one unit cell of the MnPc-3 nanotube are all bonded to one CO molecule, the spin-down conducting channels are shut down completely and the MnPc-3 nanotube is changed drastically from a half-metal to a spin-polarized semiconductor with a magnetic moment of 3.0 μ_B_ per unit cell. This shows an efficient way to tailor the electronic properties of MnPc nanoutbes: the spin state and transport properties of MnPc nanotubes can be tuned precisely by controlling the CO coverage.

**Figure 4.**
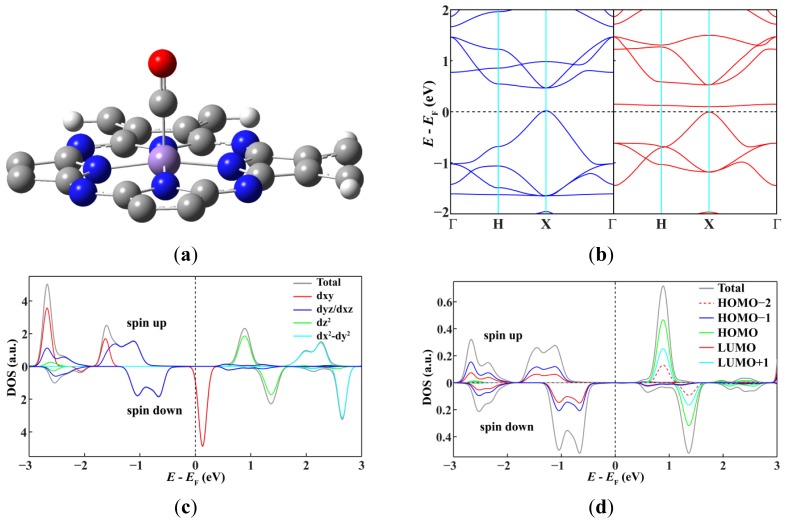
The optimized geometry structure of one unit cell (**a**) and the spin-resolved band structure (**b**) of the 2D MnPc sheet adsorbed with one CO molecule, and DOS projected onto the Mn 3d atomic orbitals (**c**) and frontier molecular orbitals of CO (**d**).

Because the curvature does not change the spin state of the Mn ions, we can analyze the mechanism of tuning the spin state of the Mn ions by using a simpler model, *i.e.*, the adsorption of CO on the 2D MnPc sheet. As shown in Figure 4, CO binds to the Mn ion of the 2D MnPc sheet in a linear configuration, giving a binding energy of 1.08 eV. The strong interaction between the Mn ion and the CO molecule leads to a redistribution of electrons in the energy bands dominated by the Mn 3d atomic orbitals resulting a change of the spin state. DOS projected onto the Mn 3d atomic orbitals shows that adsorption of CO empties the spin-up energy band dominated by the Mn 3*d*_z^2^_ orbital and shifts it to 0.77 eV above the Fermi level, leaving the spin-up energy bands dominated by the Mn 3*d*_xy_, 3*d_xz_* and 3*d_yz_* orbitals still below the Fermi level. In contrast, adsorption of CO fills completely the spin-down energy bands dominated by the Mn 3*d_xz_* and 3*d_yz_* orbitals and thus moves them from around the Femi level to more than 0.62 eV below the Fermi level. Thus, the magnetic moment per unit cell of the 2D MnPc sheet adsorbed with one CO molecule is only 1.0 μ_B_. Considering that the highest occupied molecular orbital (3σ) and the lowest unoccupied molecular orbital (1π*) of CO respectively make contributions to the DOS peaks located above and below the Fermi level, we can conclude that the interaction between the CO molecule and the Mn ion in the MnPc-related structures is a typical metal-ligand bond which can be described in terms of donation of electron density from occupied molecular orbitals of CO and back-donation from the Mn 3d orbitals with appropriate symmetry and energy into unoccupied molecular orbitals of CO [29].

We have studied the electronic and magnetic properties of single-walled MnPc nanotubes using DFT calculations, and found that all of the MnPc nanotubes investigated here are half-metals and thus can produce 100% spin polarization around the Fermi level. As the tube diameter is increased, the number of spin-down energy bands of MnPc nanotubes is always increased while the spin-up band gap of MnPc nanotubes approaches that of the 2D MnPc sheet in an oscillatory manner. Because the half-metallic character of MnPc nanotubes is deeply rooted in the distribution of electrons in the energy bands dominated by the Mn 3d atomic orbitals, fine tuning of the spin state and electronic transport properties of MnPc nanotubes can be realized by adsorption of CO molecules on the Mn ions. Furthermore, other ligand molecules such as nitric oxide, pyridine and ammonia may also be able to tailor the spin state of the MnPc nanotubes, which will be pursued in future studies. Therefore, single-walled MnPc nanotubes appear as promising candidates for future molecular spintronic devices and gas sensing devices.

## Figures and Tables

**Table 1.** Typical parameters characterizing the atomic and electronic structures of MnPc nanotubes, where D is the Mn-Mn distance perpendicular to the tube axis; Es is the strain energy; Eg is the spin-up band gap; N is the number of spin-down energy bands across the Fermi level and M is the magnetic moment per unit cell.

	**D (Å)**	**Es (meV/atom)**	**Eg (eV)**	**N**	**M (μ_B_)**
MnPc-3	8.642	35	0.63	5	9.0
MnPc-4	9.527	20	0.41	5	12.0
MnPc-5	9.949	13	0.48	7	15.0
MnPc-6	10.179	8	0.33	9	18.0
